# Pedagogical Implementation of Directive Feedback Manikins on Cardiopulmonary Resuscitation (CPR) Competencies: Expert Versus Peer Coaching

**DOI:** 10.7759/cureus.65181

**Published:** 2024-07-23

**Authors:** Jeffrey L Pellegrino, Anna Back, Ada Chan, Joanna Muise

**Affiliations:** 1 Disaster Science and Emergency Services, The University of Akron, Akron, USA; 2 Health Education Solutions, Canadian Red Cross, Ottawa, CAN

**Keywords:** ilcor, lay responder, pedagogy, directive feedback, cpr education

## Abstract

Background: Directive feedback manikins in resuscitation training evolved faster than the pedagogical evidence. Educators and learning systems must seek clarification on the efficacy of this technology to have evidence-based practices. This project explores directive feedback device use in cardiopulmonary resuscitation (CPR) education for laypersons.

Methods: A prospective nonrandomized-controlled design assessed two pedagogical approaches of directive feedback manikins in adult CPR lessons. The 230 participants were distributed between three groups: a control group without directive feedback manikins (no lights, NL), an expert coaching (EC) group with directive feedback and educator interpretation, and a peer coaching (PC) group with directive feedback, peer interpretation, and expert quality assurance.

Results: From the 25 courses observed, average compression depth (mm) did not differ between groups (*p* = .498), average compression rate (compressions: minute) significantly differed between groups (*p* = .004), and correct hand placement did not differ between groups (*p* = .249). A chi-square test showed no significant association between groups and CPR skill feedback, or between groups and "recommending the course to a friend or family member." The PC group was more likely to agree that they could “coach someone to do CPR skills” than the NL or EC.

Conclusions: This study expands the knowledge base of directive feedback manikins in a pedagogical setting to improve CPR competencies. Training organizations may consider any of these practices effective, choosing those that align with desired outcomes. CPR educators need orientation to feedback devices as well as professional development on educational options for their use. Considerations for further research include technology costs, access, and cultural aspects of implementing these tools.

## Introduction

Cardiac arrest poses a substantial health burden on individuals and society, with high nationwide mortality rates and morbidity, including profound and irreversible neurologic injury and functional disability. This in turn costs society in terms of productive years of life lost due to death or neurologic disability in the United States [1]. In public cardiac arrests, lay responder actions to recognize and respond are associated with improved health outcomes [2]. To promote the quality, efficiency, and efficacy of cardiopulmonary resuscitation (CPR) education, new technologies support the practices of educators and learners, like directive feedback manikins.

Through a lens of evidence-based practice, informed by (1) the best available research, (2) expertise in the field, and (3) public values to select, adapt, and implement interventions [3-5], we sought to compare two pedagogical approaches to directive feedback manikins. From previous evidence, directive feedback manikins (e.g., active or corrective) provide real-time, specific guidance on CPR performance, including compression rate, depth, hand position, and recoil to learners and educators. Learners can then self-correct their technique immediately during practice or educators can coach students based on feedback. In contrast, passive feedback manikins offer more basic feedback, such as a clicking sound or light indicator when a certain compression depth is reached, providing less detailed information on CPR quality. Research has shown that active/directive feedback devices positively affect CPR quality and learning outcomes, with students perceiving device-based feedback as more objective and acceptable compared to educator feedback, leading to improved skill acquisition and retention in CPR training [6-9].

As recognized resuscitation field experts, the International Liaison Committee on Resuscitation’s Education, Implementation, and Teams Taskforce recommended using directive feedback manikins in training in 2015 and 2020. They also identified a knowledge gap stating that “it remains unclear how best to use these devices, how they interact with instructors, and how timing of feedback may impact learning and retention” [7,8]. Timing might include directive feedback during practice versus testing or implementation into mastery learning versus distributive learning practices.

With the technology available for directive feedback and clinical experts in favor of the concept, public end users being “educators” (e.g., instructors or trainers) and “learners” must be included for evidence-based practice development. The American Heart Association Guidelines indicate that "corrective" feedback devices promote CPR skill acquisition and potential retention [9]. Using these devices also theoretically impacts other outcomes of learners’ confidence or intention to act, both valued outcomes in prevention and public health education [10].

Previous recommendations have not commented on or put forward how directive feedback manikins must or should be employed in the classroom to achieve the desired outcomes. An andragogical approach [11], emphasizing adult learners' need for self-directed, experiential learning with immediate application, fits a learner-centered nonschool setting of most CPR courses [12-13]. By providing real-time audio and visual feedback on compression rate, depth, and hand position, directive feedback manikins allow adult learners to correct their technique during practice, aligning with andragogical principles of active participation and problem-centered learning. Because these manikins are used within a classroom course and not independently (i.e., self-learning), there are options on how to employ the manikins in a group setting. The expert coaching (EC) option capitalizes on the educator providing corrective direction based on the feedback observed. Alternatively, peer coaching (PC) capitalizes on other learners in the course to provide corrective directions based on the feedback observed. Both approaches theoretically lower the cognitive load on the person learning the skills as they don't have to interpret and correct but only develop the psychomotor memory of the skills. PC may also extend learning opportunities by engaging more learners in the process versus passively observing while others practice.

Our objective was to provide evidence-based practice recommendations for the pedagogical implementation of directive feedback manikins when developing CPR competencies. Examined factors considered the evidence-based practice process of asking a pedagogical question; collecting data; critically appraising the evidence for its validity, importance, and applicability; and integrating evidence with practical expertise, learner values, and circumstances [14]. The guiding questions from the context of adult learners (aged 18+) of layperson CPR: (1) Does using directive feedback manikins with EC or PC improve the quality of CPR compressions (rate, depth, hand location) over manikins without directive feedback? We hypothesized learners using directive feedback manikins perform higher-quality CPR following training. (2) Does using directive feedback manikins with EC or PC improve the intention to help during cardiac arrest over manikins without directive feedback? We hypothesized learners using directive feedback manikins would have a higher Intention to Aid (I2A) score than users who learn without directive feedback manikins, and learners engaging as peer coaches while using feedback manikins will have a higher I2A score than those users who learn from expert coaches with or without directive feedback manikins [15]. (3) As consumers, do learners appreciate the experience of using directive feedback manikins with EC or PC? We hypothesized learners using directive feedback manikins will be more satisfied and likely to recommend training at a higher rate than those who learn without directive feedback manikins.

## Materials and methods

We prospectively assessed the learning outcomes of lay responder adults between two interventions using directive feedback and a control group to understand pedagogical implementation. We assessed knowledge and attitudes within and between groups using the I2A tool based on the Theory of Planned Behavior, surveying elements of attitudes, social norms, and confidence [16]. I2A includes five knowledge questions to differentiate learners but does not seek to assess all knowledge (Table 1). Skills were assessed by CPR educators for certification purposes and by a recording manikin (Brayden Pro App and associated Bluetooth-ed Brayden Pro manikins (https://www.innosonian.us/brayden-pro/) for study data.

**Table 1:** Knowledge questions from the I2A survey CPR: Cardiopulmonary resuscitation; AED: automated external defibrillator Source: Intention to Aid (I2A) questionnaire [15]

Knowledge question text	Style of question
A person who is not responsive and gasping air irregularly or making strange noises should be given CPR	True or false
Click (highlighting green) the correct position to place the AED pads	Picture of a supine person with five hidden “hot spots,” the participant clicked on the picture in the appropriate area
When giving chest compressions to an adult, you should push down at least _____	Multiple choice
Click the box (highlighting green) where you place your hands for chest compressions during CPR	Picture of a supine person with five hidden “hot spots,” the participant clicked on the picture in the appropriate area
Does the law protect you while you "help" someone, as long as you act appropriately?	True or false

**Figure 1:**
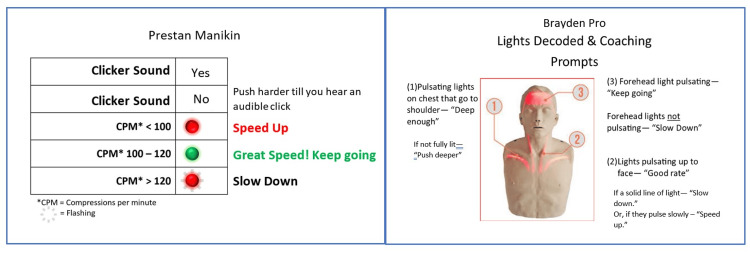
Peer coaching card samples CPM: Compressions per minute Image content adapted with permission from Prestan Products^(R)^ and Innosonian

A nonrandomized, controlled field-based design allowed the observation of the use and outcomes of two pedagogical approaches. The EC intervention utilized a directive feedback manikin with educators interpreting lights and coaching skill performance. The ratio of expert to learner was 1:12. The PC intervention utilized the same manikin, with an approximately four-minute orientation for learners to the feedback lights and coaching prompts. Learning pairs had a coaching card to remind them of light meanings and appropriate prompts during the skill session (Figure 1). A control group (no lights, NL) used manikins without directive feedback engaged. Educators independently verified that all skills were compliant with the curriculum’s specifications.

CPR educators certified by the Canadian Red Cross and St. John Ambulance Canada were eligible to participate. In September 2021, 13 volunteers underwent a one-hour orientation to the purpose, protocols, data collection logistics, and provided PC cards. Due to COVID-19 logistical complications, only four educators submitted data. Each educator was asked to record seven lay responder adult CPR segments (15-30 minutes) within any CPR or first aid course in the following sequence: one control course (NL), three EC, and three PC using a directive feedback manikin. The NL course allowed data collection on the standard practice and norm of the data collection process for the EC and PC interventions. From March 2022 to April 2023, 25 courses were observed. Learners self-selected into a course location and date; they were unaware of the study or which group they were assigned to. Groups of homogenous learners (comprised of members of the same organization) were not included. Course completion criteria remained the same across the three arms.

CPR learners, after consenting to participate, started the I2A survey by responding with demographics and previous CPR experience. Post-training CPR learners were asked to provide two minutes of compression-only CPR in which compression depth (mm), compression rate (per minute), and correct hand position (%) were recorded. Participants then completed the I2A and a brief survey of their experience. No monetary compensation was offered. The University of Akron Institutional Review Board approved this research, #20210413, and we adhered to the Guideline for Reporting Evidence-based practice Educational interventions and Teaching (GREET) [17].

I2A data and CPR knowledge scores were generated using QualtricsXM© (2023) (Qualtrics, Seattle, USA). I2A results were analyzed using the Winsteps® Rasch measurement computer program (Version 4.5.5) (Winstep Software Technologies). Group comparisons were calculated using IBM SPSS Statistics for Windows, Version 28 (Released 2021; IBM Corp., Armonk, New York, United States). A chi-square test of independence was performed to evaluate the relationship between the three groups and categorical survey questions and analysis of variances (ANOVAs) for continuous variables. All statistics were considered with an alpha of <.05 to be significantly different.

## Results

CPR learners

A total of 230 eligible CPR learners agreed to participate (*n* = 230) out of 254 enrolled in courses (90.6%). The demographics are described in Table 2 regarding gender, prior CPR experience, recency of previous training, and the primary reason for attending. Using a Pearson chi-square (*x^2^) *analysis across the three arms, gender, number of previous courses, and the primary reason for taking the course were shown to be similar. A sub-analysis revealed a higher percentage of employment-required reasons in the EC group (74.2%) than in the NL or PC groups (59.1% and 59.9%, respectively). The PC group had a significantly higher percentage (30.9%, *probability value* (*p*)* *= .037) of first-time learners.

**Table 2:** Demographics of CPR learners *Significant difference, based on adjusted residual ≥ 1.9 (95th percentile); *n*:number of participants; CPR: cardiopulmonary resuscitation

	Control (*n *= 23)		Expert coaching (*n *= 124)		Peer coaching (*n *= 83)		Overall (*n *= 230)	
	n	%	n	%	n	%	n	%
Gender								
Female	6	27.3	43	36.4	28	35.0	77	33.5
Male	16	72.7	74	62.7	52	65.0	142	61.7
Trans	0	0.0	1	0.8	0	0.0	1	0.4
Missing/other/not answered	-	-	-	-	-	-	10	4.4
Previous CPR courses								
First course	3	13.6	19	15.7	19	23.8	41	17.8
One course	5	22.7	20	16.5	12	15.0	37	16.1
Two courses	5	22.7	17	14.0	8	10.0	30	13.0
Three courses	3	13.6	17	14.0	11	13.8	31	13.5
Four courses	1	4.5	10	8.3	5	6.3	16	7.0
Five+ courses	5	22.7	38	31.4	25	31.3	68	29.6
Missing/no response	-	-	-	-	-	-	7	3.0
Most recent CPR course (years prior)								
None	0	0.0	12	13.0	17	30.9*	29	12.6
Within validation period (three years)	8	50.0	40	43.5	17	30.9	65	28.3
Within one guideline period (five years)	6	37.5	21	22.8	13	23.6	40	17.4
More than one guideline period (six+ years)	2	12.5	19	20.7	8	14.5	29	12.6
missing/no response	-	-	-	-	-	-	67	29.1
Primary reason for taking course								
Work required	13	59.1	89	74.2*	48	59.9	150	65.2
Personal interest	2	9.1	15	12.5	17	21.3	34	14.8
Care for family/friend	0	0.0	0	0.0	1	1.3	1	0.4
Other	3	13.6	11	9.2	6	7.5	20	8.7
Combination of reasons	4	18.2*	5	4.2	8	10.0	17	7.4
Missing/no response	-	-	-	-	-	-	8	3.5

Learners came from the Canadian Red Cross (18 courses, number in group (*n)* = 177) and St. John Ambulance Canada (seven courses, *n* = 53). The gender distribution was 61.7% male, with no significant difference between arms (*p* = .785). Almost 30% had five or more previous CPR courses, and approximately 21% had their first CPR course. Of those reporting previous courses, almost 30% reported taking their last course more than five years prior, with no significant differences between arms (*p* = .834). Participants were allocated by group: 23 (10%) participants in NL, 124 (53.9%) EC, and 83 (36.1%) PC.

In examining CPR competencies, the 23 NL participants (mean (*M*) = 91.3; standard deviation (SD) = 10.14) compared to the 207 with directive feedback (*M* = 82.6; SD = 16.8) demonstrated significantly higher knowledge scores, t-statistic (t)(228) = 2.43 and *p* = .016. The effect size, as measured by Cohen’s *d* [18], was *d* = 0.63. A significant effect was noted (*f-statistic (F*) (2,57) = 7.696; *p* < 0.001) between the three arms; in a post hoc analysis, EC was significantly lower than both NL and PC groups (*p* = .008 and .011, respectively). The very small effect size, calculated as eta squared (η²) 0.06.

Three psychomotor skills were assessed independently using one-way ANOVA. Average compression depth (mm) did not differ between groups (*p* = .498); see Table 3 for means and standard deviations (*p* = .498). The average compression rate (compressions: minute) significantly differed between groups (*p* = .004), with a post hoc analysis showing the difference between the NL and the EC (*p* = .004). The correct-hand placement score did not differ between groups (*p* = .249).

**Table 3:** Competency outcomes by group p: Probability; SD: standard deviation; mm: millimeter; ANOVA: analysis of variance *Statistically significant at < .05

	Control group		Expert coaching		Peer coaching		Overall		ANOVA (p)	Effect size (eta-squared)
	Mean	SD	Mean	SD	Mean	SD	Mean	SD		
Knowledge	91.30	10.14	79.84	18.30	86.75	13.35	83.48	16.46	.001*	0.063
Skills										
Average compression depth (mm)	51.79	5.03	54.15	7.09	52.29	7.30	52.75	6.56	.498	0.024
Average compression rate (minute)	106.95	12.83	119.10	10.48	112.52	9.03	112.95	11.74	.004*	0.178
Overall correct hand position (%)	94.53	15.51	94.60	14.02	100.00	0.00	96.47	11.98	0.249	0.048
Intention to aid person measure	51.02	1.54	50.73	1.54	51.09	1.41	50.89	1.50	0.212	0.014

Affectively, the 23 NL participants (*M* = 51.02; SD = 1.54) compared to the 207 with directive feedback (*M* = 50.88; SD = 1.49) did not significantly differ in their I2A in an emergency, *t*(228) = 0.419; *p* = .675). The I2A score was assessed between groups by using a one-way ANOVA. The NL (*M* = 51.02; SD = 1.54), EC (*M* = 50.73; SD = 1.54), and PC (*M* = 51.09; SD = 1.41) did not differ between groups (*p* = .212).

Participants were asked about their level of agreement with the statement “I received appropriate feedback on my CPR skills” using a five-point Likert scale from strongly agree to strongly disagree. The overall percentage of those who “somewhat agreed” or “strongly agreed” was 95.6% (NL= 95.7%; EC= 95.8%; and PC= 95.1%). A chi-square test showed no significant association between groups and CPR skill feedback: *x^2^*(8, *N* = 224) = 11.54; *p* = .17.

A second Likert agreement question was asked regarding “I could coach someone to do CPR skills.” The overall percentage of those who “somewhat agreed” or “strongly agreed” was 85.9% (NL= 86.9%; EC= 80.2%; and PC= 93.8%). Based on a chi-square test, the relationship between groups was significant: *x^2^*(8, N = 219) = 18.54; *p* = .02. The PC group was more likely to agree that they could “coach someone to do CPR skills” than the NL or EC.

Finally, learners were asked to “Recommend this course to a friend or family" using a 10-point scale from “Would not recommend” (1) to “Will recommend” (10). Out of the 187 responses, two (1.1%) were below the midpoint/neutral (5) and two were at the midpoint/neutral (1.1%) overall. The majority reported they “will recommend” (10) or (9), NL (80.9%), EC (74.2%), and PC (73.9%). A chi-square test showed no significant association between groups and recommending the course to a friend or family member: *x^2^*(14, *N* = 187) = 9.50; *p* = .80.

## Discussion

Expected versus observed results

The triangulation of objective measurements, I2A, and consumer feedback provides evidence for training organizations to consider using EC or PC with directive feedback manikins when facilitating lay responder CPR courses. Objectively, the three arms all performed on average within the recommended guidelines of 5-6 mm for compressions and 100-120 compressions per minute [19]. Correct hand position percentage was also high (<90%) across the groups, with the highest (100%) in the PC group. This difference may be explained by more time and attention to tasks during peer engagement. This information is valuable because it shows which aspects of CPR performance might be improved with different uses of feedback devices.

Knowledge scores favored the control group, a possible artifact of the number of recently trained members, or it could imply that directive feedback devices, while useful for CPR skill development, may not necessarily lead to better overall knowledge scores. The I2A scores were similar between groups, ranging from 50.73 to 51.09. We hypothesized that PC would be higher because the additional time on task could build more self-efficacy, but this did not appear in the data. This suggests that different feedback device methods don't significantly change how likely someone is to step in and help during an emergency.

The third element of triangulation looked beyond a skill or knowledge set to gain insight into what the class meant for their future decisions. Learners "agreed" to "strongly agreed" that they received appropriate feedback in each group (95.6%). The PC group reported significantly higher agreement with the statement "they could coach someone to do CPR skills," which is supported by the tools and practice they had during the course. This data suggests that most participants felt confident they could coach someone else in CPR; however, the type of engagement with feedback devices did make a difference.

The global question of recommending the course to a family member or friend showed no statistical difference between groups. This data suggests that almost all participants, regardless of their training group, would recommend the CPR course to friends or family. The type of feedback device engagement did not significantly affect how likely participants were to recommend the course. This indicates that all versions of the course were well-received by participants. Although we were curious as to any gender preferences, we found no outcome differences within groups by gender or when recommending the course.

Learning system considerations 

Evaluating multiple learners simultaneously presents challenges to measuring consistently and objectively. Learners were engaged differently in the interventions with the educator, removing one degree from corrective feedback in PC, allowing for potentially deeper learning for educators to spend more time observing, or reducing the educators' cognitive load. Costs may include a reliance on technology by the educator, resulting in a potential loss of skill when technology is available. From a learning outcome perspective, directive feedback devices are enablers for what others could perceive as objective assessments. Serendipitously, they may promote confidence in supervisors, training organizations, authorities, and the public that a standard is upheld.

The pandemic challenged educators to shift, often incorporating new technology and requiring a minimum level of digital literacy among learners and educators [20]. This study introduced logistical and methodological considerations to ease educators' adoption, implementation, and habituating use of feedback devices. It is unknown whether educators teaching outside the study would be afforded the same time and support and how learners with limited digital literacy might perceive the tool.

We did not observe or calculate the time added or substituted in a course for orientation to the feedback system. We estimated, from pilot courses, 2-4 minutes based on the number of questions asked by learners. One educator commented that the use of any directive feedback intervention is based on the context of the learners; for example, PC might be helpful with previously trained learners to engage and enhance existing skills. We also did not measure any additional cognitive load, as suggested by Cheng et al. [9] on the educator or learner. We hypothesize that in the EC group, the cognitive burden is placed on the educator and that in the PC group, it is on the peer coach and not the compressor in either case. A novice educator could also have an additional cognitive load with PC. The diversity of any class could use EC, PC, or both, allowing learners and educators to focus on learning outcomes.

Limitations

This project was initiated in 2019 as an in-person, educator-led approach, within a field setting to increase external validity. Although external validity increased, there were internal validity limitations that occurred because of the pandemic, curtailing the selection of CPR educators (recruited persons leaving the field for alternative employment) and courses due to government and educational organization policies for holding face-to-face learning. Collecting a sufficient sample from our remaining educator sample was challenging. Recording one control (NL) course for each educator may have led to an undersampling of this group; however, this is the current standard and allowed us to collect more data on the EC and PC interventions.

This study contributes to the pedagogical affordances of directive feedback devices in face-to-face courses but did not consider directive feedback devices in spaced learning. Although CPR course learners participated, their diversity of experience may have diluted the efficacy for any subgroup, like first-time learners or gender. Additional research is needed to address how directive feedback devices could be applied when learning at a distance (educator-led virtual learning) or in asynchronous contexts. Additionally, this study considered devices from only two brands. Finally, this inquiry did not seek to measure if directive feedback allowed people to complete course requirements faster and more accurately or recall competencies for longer, which could be follow-up studies.

Recommendations

Educators must develop competencies to coach without directive feedback devices. After this competency develops, the layering-in of feedback devices provides additional tools to meet learner and class-level pedagogical needs. Training organizations should develop training and resources, such as coaching cards, checklists, and facilitation guides that support educators’ integration of devices, along with instructional techniques of PC and EC, and how to interpret feedback. Training agencies will also need to orient educators to the use and pedagogy of different devices as technology and pedagogy improve.

## Conclusions

We assessed the CPR learning outcomes of adult lay responders who experienced EC or PC while using directive feedback manikins. Of the three psychomotor skills measured, only the compression rate significantly differed between the control and EC groups (the average compression depth and overall hand placement did not differ between groups). While the PC group was more likely to “coach someone to do CPR skills” than the control and EC groups, the I2A scores and satisfaction with the course were similar between all groups, suggesting that different feedback device methods do not significantly change how likely someone is to help during an emergency or recommend a course. This project expands the knowledge and application of directive feedback devices. Educators who are competent in EC and PC may benefit from employing either or both within a given learner group based on learner characteristics and resources. 
